# Brain Injury after Transient Global Cerebral Ischemia and Subarachnoid Hemorrhage

**DOI:** 10.1155/2013/827154

**Published:** 2013-11-12

**Authors:** Fatima A. Sehba, Ryszard M. Pluta, R. Loch Macdonald

**Affiliations:** ^1^Departments of Neurosurgery and of Neuroscience, Mount Sinai School of Medicine, New York, NY 10029, USA; ^2^Surgical Neurology Branch, National Institute of Neurological Disorders and Stroke, National Institutes of Health, Bethesda, MD 20892, USA; ^3^Division of Neurosurgery, St. Michael's Hospital, Labatt Family Centre of Excellence in Brain Injury and Trauma Research, Keenan Research Centre of the Li Ka Shing Knowledge Institute of St. Michael's Hospital, Department of Surgery, University of Toronto, Toronto, ON, Canada M5B 1W8

Brain injury of diverse etiology is frequently encountered clinically. Management, thus, is diverse. Transient global ischemic (TGI) brain injury may result from cardiac arrest where cerebral perfusion diminishes to the point that blood supply can no longer meet the metabolic demand of the brain or from aneurysmal subarachnoid hemorrhage where hemorrhage from an intracranial aneurysm elevates the intracranial pressure above the blood pressure leading to momentary perfusion arrest.

Though there are commonalities and differences in the etiology and pathogenesis of these two brain injuries, whether they required differential management or they can be grouped together is not clear. Some risk factors of TGI and SAH are shared and common and others are unique to the injury. Shared risk factors include high blood pressure, smoking, alcohol abuse, and stress. The risk factors unique to TGI are clinical conditions such as cardiac arrest (major cause), shock that creates prolonged hypoxia or hypoglycemia, pathologically elevated cerebral metabolic rate, or decreased cerebral perfusion pressure. The risk factor unique to SAH is the presence of an intracranial aneurysm. Other contrasting risk factors are age and gender; whereas old age increases the risk of TGI, SAH occurs in a younger population with an average age of 52–55 years. Women harbor significantly more intracranial aneurysms than men and consequently are more frequently the victims of SAH. Although the average age of women with SAH is greater than men, the outcome is similar [1]. In contrast, more men than women are at risk of ischemic stroke and women with ischemic stroke are usually older and more likely to die of stroke than men [2].

Brain injury after TGI can be separated into an initial ischemic phase that lasts for the duration that the brain blood supply remains reduced (usually ≤10 minutes, otherwise death is inevitable) and the reperfusion phase that begins immediately after reconstitution of cerebral blood supply (>10 minutes). The initial phase of brain injury after SAH is more complex and lasts for 48 to 72 hours. A complex series of events occurs during this initial (early) phase, including blood-induced mechanical trauma, oxyhemoglobin (released upon degradation of blood) induced oxidative stress, inflammation, and ischemia [3]. A delayed phase of brain injury, unique to SAH, develops 3–7 days after SAH. This injury is characterized by angiographic vasospasm and delayed cerebral ischemia [4].

In order to help understand the management of brain injury after TGI and SAH, this special issue compares and contrasts the various mechanisms of brain injury after TGI and SAH. It presents two original research articles and 7 reviews. The research article by C. S. Jung et al. studies the correlation of serum and CSF injury markers with ischemic events in SAH patients and that by S. O. Eicker et al. compares neuroprotective qualities of vascular endothelial drive growth factor (VEGF) against stroke and cerebral vasospasm after SAH. F. A. Sehba and R. M. Pluta review the existing TGI and aSAH animal models and present a modified aSAH model which effectively mimics the disease and has the potential of becoming a better resource for studying the brain injury mechanisms and developing a treatment. M. A. Kamp et al. review the mechanisms and clinical significance of the alteration in calcium and potassium channel after SAH and TGI. M. K. Tso and R. L. Macdonald review preclinical studies on microvascular changes and their therapeutic modification following SAH and TGI. N. Plesnila compares and contrasts pathophysiological events occurring in experimental models of SAH and TGI and evaluates the contribution and importance of global cerebral ischemia in the pathophysiology of SAH. M. Koide et al. summarize the current knowledge regarding the impact of SAH and global ischemia on neurovascular communication. J. A. Frontera reviews clinical trials in cardiac arrest and SAH and concludes that clinical trials in SAH assessing acute brain injury are conducted and that these trials may receive benefit from interventions identified successfully against brain injury following cardiac arrest.

We hope that the present special issue stimulates further research in this topic and brings to attention the information that would provide a better understanding of the management of TGI and SAH patients.

*Fatima A. Sehba* *Fatima A. Sehba* *Ryszard M. Pluta* *Ryszard M. Pluta* *R. Loch Macdonald* *R. Loch Macdonald*
